# Comment on: Evaluating the impact of nitisinone at mosquito-lethal doses on *Lutzomyia longipalpis*

**DOI:** 10.1371/journal.pntd.0014096

**Published:** 2026-07-02

**Authors:** Kristopher Hawkins, Lee Rafuse Haines, Álvaro Acosta-Serrano

**Affiliations:** 1 Department of Biological Sciences, University of Notre Dame, Notre Dame, Indiana, United States of America; 2 Eck Institute for Global Health, University of Notre Dame, Notre Dame, Indiana, United States of America; London School of Hygiene and Tropical Medicine, UNITED KINGDOM OF GREAT BRITAIN AND NORTHERN IRELAND

Nitisinone, a β-triketone herbicide, is a well-characterized inhibitor of tyrosine metabolism in humans. In the early 2000s, it was repurposed and approved by the FDA for the treatment of infants born with tyrosinemia type I [1], and, more recently, for individuals with alkaptonuria [2]. The drug inhibits the enzyme 4-hydroxyphenylpyruvate dioxygenase (HPPD), leading to the accumulation of upstream metabolites, primarily tyrosine and phenylalanine. Given that tyrosine metabolism is essential for blood-feeding arthropods [3], several research groups have successfully investigated nitisinone and other HPPD inhibitors as potential tools for vector control.

Augendre et al. [4] recently reported on the susceptibility of the sand fly vector of visceral leishmaniasis in the Americas (*Lutzomyia longipalpis*) to nitisinone. To date, nitisinone has been shown to be effective against multiple vector species, including mosquitoes [3,5–7], tsetse flies [8], bed bugs [9], kissing bugs [3], and ticks [3,5], making its evaluation in other medically important vectors such as sand flies both timely and highly relevant, with clear potential for development as a pan-vector control strategy. However, several aspects of the experimental design, dose selection, and data interpretation substantially limit the strength of the conclusions.

In the study, sand fly susceptibility was assessed using a single dose (250 ng/mL, 0.76 µM) delivered in a blood meal, chosen based on midpoint lethality in mosquitoes rather than on sand fly–specific dose–response data. While cross-taxon comparisons can be informative, nitisinone lethality is known to depend strongly on species, exposure route, and administered concentration. For example, in *Glossina morsitans morsitans*, nitisinone delivered via an artificial blood meal caused rapid mortality with an LC50 of 682 ng/mL (2.07 µM) at 24 hours, and survival outcomes were explicitly modeled as a function of dose [8]; topical application also induced rapid mortality but with a different LC50 (39 pmol/fly) [8]. Similarly, feeding experiments with nitisinone-containing human blood demonstrated concentration-dependent mosquitocidal activity against *Anopheles gambiae*, including insecticide-resistant strains and older cohorts [6]. In this context, the absence of a positive control in the study by Augendre et al. [4], using a clearly lethal nitisinone concentration, is a critical limitation, as exposure to the drug is inferred rather than demonstrated. Inclusion of such a control would have established assay sensitivity, verified compound bioavailability, and distinguished true biological resistance from experimental underdosing.

Against this background, the reliance on using a single mosquito-derived midpoint concentration fundamentally limits the interpretability of the sand fly findings. The results are presented primarily as “p > 0.05,” with their sole figure caption (see Fig. 1 from Augendre et al. [4]) noting a log-rank comparison, but without reporting hazard ratios, confidence intervals, censoring details, or a meaningful assessment of statistical power. This limits the interpretability of the survival analysis and makes it difficult to evaluate whether the study could detect biologically relevant effects.

Physiological differences in the sizes of blood-meals further complicate the use of mosquito-derived concentrations as a proxy for sand flies. Female *Phlebotomus papatasi* and *Lu. longipalpis* ingest blood-meal volumes of approximately 0.9 µL [10]. By contrast, female *An. gambiae* ingest substantially larger blood meals, typically ranging from 2.5 to 4.5 µL, with median values near 3 µL [11]. Because the lethality of nitisinone depends on the total amount ingested and the digestion rate rather than nominal blood-meal concentration alone, applying a mosquito-derived nitisinone concentration without adjusting for sand fly blood-meal size and feeding physiology is expected to result in inadequate dosing. Under these conditions, the reported absence of mortality is most plausibly explained by insufficient dose rather than sand flies’ innate tolerance of nitisinone.

Interpretation is further limited by insufficient detail on how the dose was delivered to the sand flies. Although the study reports initial cage sizes of approximately 150 unfed female sand flies, it does not report the proportion of insects that successfully blood-fed, the volume of blood ingested per female, or the distribution of fed versus unfed flies (feeding success). This makes it impossible to assess effective sample size, exposure heterogeneity, or statistical power. This limitation is particularly relevant given their rearing and feeding conditions. Flies were maintained on a 50% organic sugar solution, followed by only a 24-hour starvation period prior to blood feeding. Prior access to sugar has been shown to alter blood-feeding behavior and reduce feeding avidity in mosquitoes [12], and residual sugar meals have been associated with reduced blood-feeding frequency [13]. When coupled with a brief starvation period (one day), these conditions are likely to simultaneously reduce feeding success and increase variability in the size of blood-meals ingested, which further obscures the relationship between administered concentration and effective dose received. In essence, a sand fly with residual sugar in its crop is likely to ingest a smaller volume of blood, thereby further reducing the dose of nitisinone it receives.

Furthermore, this study did not verify nitisinone ingestion in fed insects or assess downstream metabolites, and it did not report any surrogate markers of nitisinone uptake. In contrast, related studies in tsetse flies and mosquitoes paired survival outcomes with clearly defined dosing regimens and, where feasible, internal confirmation of ingestion and physiological effects following feeding [6,8]. Without such confirmation in sand flies, it remains uncertain whether nitisinone achieved biologically relevant concentrations in target tissues.

Finally, the authors report only two independent biological replicates (see their Fig 1 [4]), without detailing how between-replicate variability was handled analytically or justifying how such limited data supports robust statistical inference. This further weakens confidence in their conclusions.

In closing, the nitisinone concentration evaluated by Augendre *et al*. in sand flies is well below experimentally established lethal thresholds for other vectors and was applied under conditions that likely limited effective dose delivery. When considered alongside controlled dose-response data and related work in other hematophagous insects, the reported survival outcomes are consistent with insufficient exposure rather than intrinsic sand fly tolerance. Rigorous quantification of feeding, ingestion, internal exposure, and species-appropriate dose-response relationships is therefore essential before robust conclusions regarding sand fly susceptibility to nitisinone can be supported.
